# Extralaryngeal Terminal Division of the Inferior Laryngeal Nerve: Anatomical Classification by a Surgical Point of View

**DOI:** 10.1155/2013/731250

**Published:** 2013-05-30

**Authors:** Emin Gurleyik

**Affiliations:** Department of Surgery, Duzce University, Medical Faculty, 81650 Duzce, Turkey

## Abstract

*Background*. Complete anatomic knowledge including all variations of the inferior laryngeal nerve (ILN) is mandatory for thyroid surgeon. Extralaryngeal terminal division (ETD) of the ILN has significant importance for the safety of thyroidectomy. *Material and Methods*. Surgical dissection of 200 ILNs was performed on 100 cases. The presence of ETD of the nerve was determined intraoperatively. We propose by a surgical point of view a regional (segmental) classification of ETD of the ILN along its cervical course. *Results*. ETD has been observed in 54/200 nerves (27%). Great majority are bifurcated nerves (trifurcation 2%). Four types of ETD are classified. In type 1 (arterial; 46.3%), ETD has occurred near inferior thyroid artery (ITA). In type 2 (postarterial; 31.5%), division has been found on postarterial segment. In type 3 (prelaryngeal; 11%), division has been located very close to laryngeal entry point. In type 4 (prearterial; 11%), ETD has occurred before the nerve crossing the ITA. *Conclusions*. ETD of the ILN is a common anatomical variation. The bifurcation occurs in the ILN at various distances from laryngeal entry point. The classification increasing surgeons' awareness may help to simplify identification and exposure of terminal branches. Preservation of both extralaryngeal terminal branches of the ILN has paramount importance for the safety of thyroid operations.

## 1. Introduction

The safety of thyroid operations mainly depends on complete anatomic knowledge of the inferior laryngeal nerve (ILN) including all its variations. The nerve has many anatomic variations along its expected and unexpected cervical course. The important one is cervical (extralaryngeal) branching of the nerve. The presence of many branches and sensory innervations of adjacent tissues by thin branches of ILN has been reported up to 90%. These branches have been established by anatomical studies under direct or microscopic observation [1, 2]. These thin neural structures are not generally perceived during surgery of the thyroid. Therefore, these branches are important scientific knowledge by anatomical point of view but not by surgical point. On the other hand, extralaryngeal terminal division (bifurcation) of the ILN is an anatomic variation macroscopically discovered during operations along cervical course of the nerve. The terminally bifurcated nerve has usually two larger branches which have same or closer size as diameter. Larger branches of the nerve may have significant effect on function of laryngeal musculature. The thyroid surgeon must carefully expose and preserve the integrity of both terminal branches if present. Therefore, macroscopic terminal division of the nerve seems to carry great importance by surgical point of view. 

In this study, we aim to establish the rate of anatomical features and location of division along cervical course of the nerve and surgical importance of extralaryngeal terminal bifurcation of the ILN in series of thyroidectomy cases. We try to classify anatomical location of division point on the cervical course of the nerve. 

## 2. Patients and Methods

A prospective study on surgical anatomy of the ILN was conducted on 100 patients with surgical diseases of the thyroid gland between May 2009 and September 2012. Patients with reoperative surgery for treatment of recurrent diseases were not included. Total thyroidectomy is the surgical procedure in all these 100 patients. All operations were performed by a single surgeon in order to provide a standard dissection. Main subjects of this study are the presence and incidence of extralaryngeal terminal division of the nerve which are macroscopically observed. Location of this division along cervical course of the ILN is determined. Lateral sides (unilaterally right or left, or bilateral) of the terminal division are also noted during thyroid operations.

### 2.1. The Dissection Technique of ILN

 After freeing and medially mobilizing lateral lobes of the thyroid gland at both sides with classical surgical approach, the inferior thyroid arteries (ITA) were identified, isolated, and a loop of silk suture was placed around arteries for traction. With usual lateral approach, ILNs were identified below the artery and fully isolated at both sides. The nerve is carefully exposed in the trachea-esophageal groove until its laryngeal entry point. The dissection is performed using binocular loupe. If macroscopically and clearly delineated terminal divisions of the nerve are identified along its cervical course, we study anatomical features of these branches.

### 2.2. Extralaryngeal Terminal Division of the ILN

Division of the ILN occurs along its cervical course prior to laryngeal entry. The branches are macroscopically observed during thyroid operations, and they enter separately into the larynx by different points. 

### 2.3. Classification of Terminal Division of the Nerve

**Figure 1:**
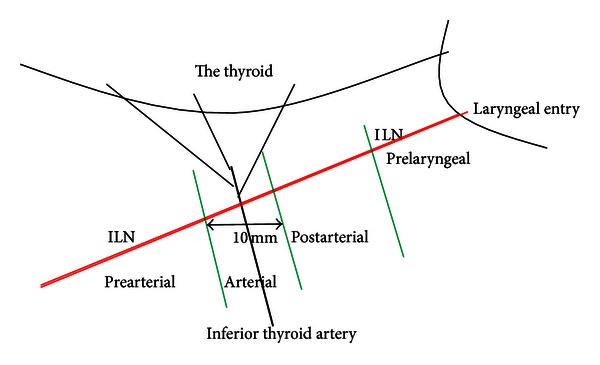
Segments in cervical course of the inferior laryngeal nerve (ILN).

We propose a regional (segmental) classification of extralaryngeal terminal division of the ILN along its cervical course. The classification is based on surgical exposition steps of the entire nerve and on the rate of different location of the bifurcation point. Risk regions for injury to the nerve are also taken into account for classification of extralaryngeal bifurcation of the ILN by surgical point of view. Regions and anatomical location of terminal division is classified basing on two relatively constant landmarks; the neurovascular (ILN and ITA) crossing point and laryngeal entry point of the nerve (Table 1; Figure 1). 

## 3. Results

Results were obtained from surgical dissection of 100 consecutive total thyroidectomy cases. Two hundred ILNs were identified, isolated, and entirely exposed along their cervical courses until the laryngeal entry. Seventy-seven percent of our patients are females. The incidence and anatomic features of extralaryngeal terminal division of the ILN are the main subject of the study.

Extralaryngeal terminal divisions of ILNs were determined in 43 (43%) patients. We observed bilateral division in 11 (11%) patients. Extralaryngeal terminal division has been macroscopically observed in 54/200 nerves (27%). The rate of division of the ILN is 19% and 35% at right and left sides, respectively (Table 2).

**Figure 2:**
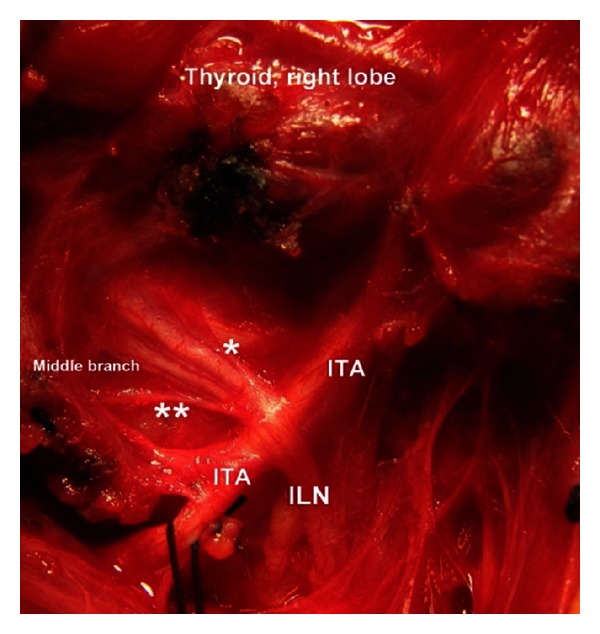
Extralaryngeal terminal *trifurcation* of the right ILN. Division of the nerve occurs at neurovascular crossing. *Anterior branch, **Posterior branch.

The ILNs have been terminally trifurcated (divided by 3 branches) along their cervical courses in 4 (4%) patients unilaterally, 2 trifurcations at right or left sides each. This result reflects an incidence of trifurcation as 2% of exposed ILNs (Table 2; Figure 2).

**Figure 3:**
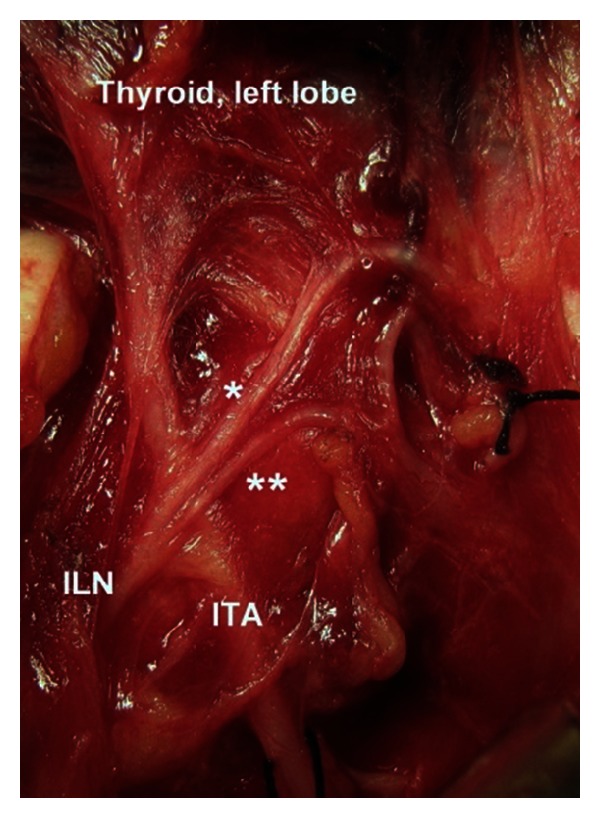
Terminal division of the left inferior laryngeal nerve (ILN) occurs at arterial segment (Type 1). *Anterior branch. **Posterior branch. ITA: inferior thyroid artery.

**Figure 4:**
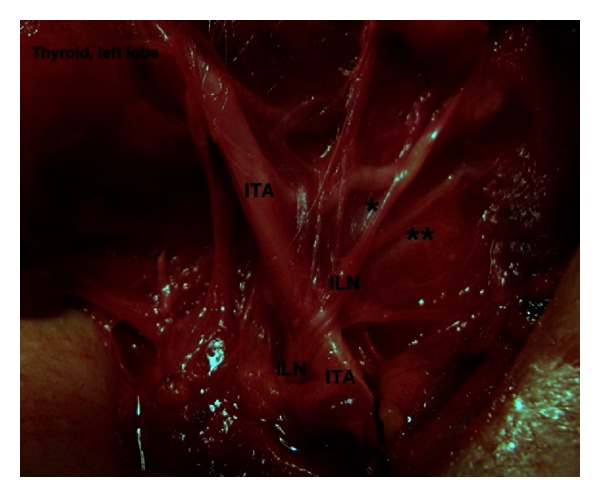
Terminal division of the left inferior laryngeal nerve (ILN) occurs at post-arterial segment (Type 2). *Anterior branch. **Posterior branch. ITA: inferior thyroid artery.

**Figure 5:**
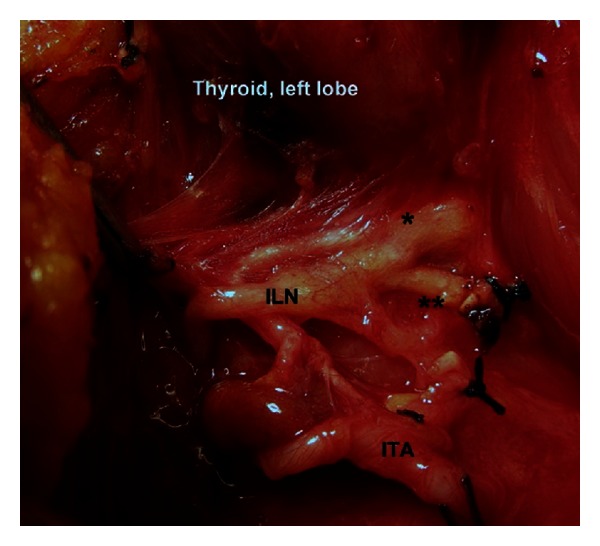
Terminal division of the left inferior laryngeal nerve (ILN) occurs at pre-laryngeal segment (Type 3). *Anterior branch. **Posterior branch. ITA: inferior thyroid artery.

**Figure 6:**
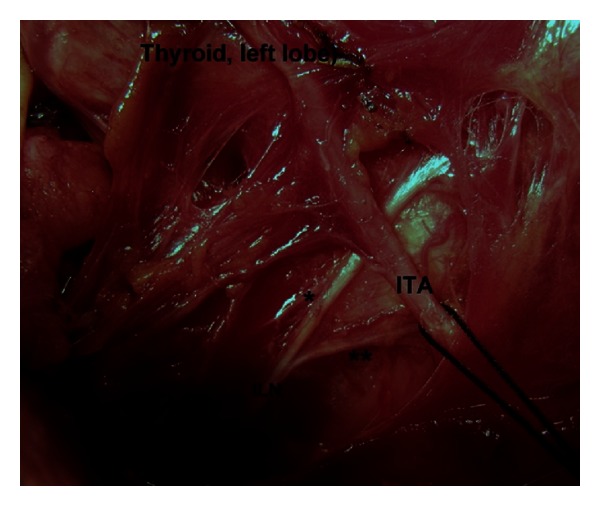
Terminal division of the left inferior laryngeal nerve (ILN) occurs at pre-arterial segment (Type 4). *Anterior branch. **Posterior branch. ITA: inferior thyroid artery.

The location of terminal division along cervical course of the nerve is another remarkable finding of the present study. The cervical course of the nerve is divided by four segments in which the neuro-vascular crossing (the ILN and the ITA) and laryngeal entry points are found as important landmarks for regional classification and location of terminal division of the nerve. Approximately half (46.3%) of the terminal division of the ILN has occurred in arterial region.  Type 1 is a more common location of division point followed by Type 2.  Type 3 (prelaryngeal) and Type 4 (prearterial) have seldom observed as location of terminal division of the ILN (Table 3). Intraoperative photos of various locations of terminal division point of the ILN show in vivo anatomic variations of the nerves (Figures 3, 4, 5, and 6). 

## 4. Discussion

 Microanatomic studies have shown that the ILN divides intralaryngeal branches to anastomose with branches of the internal laryngeal nerve. Size of these branches has been reported 0.6 mm as diameter [3]. When a surgeon macroscopically observes a single trunk nerve in its cervical course, it has intralaryngeal branches for connections of laryngeal nerves. Sometimes these branches have been exposed prior to laryngeal entry. Therefore, we can comment that extralaryngeal terminal division of the ILN is a premature branching of the nerve. By a surgical point of view, the awareness about the occurrence of premature terminal division is important that exposing bifurcation and larger branches of the nerve separately prevent nerve injury during thyroid operations. In this study, we try to increase surgeons' awareness about this anatomic variation of the ILN by anatomically classifying the distribution of branching in its cervical course. 

Complete exposure of ILN is mandatory to avoid nerve injury and laryngeal muscle palsy. Its anatomic variations threaten nerve integrity even in experienced hands. Our rates (27% of ILNs) of macroscopically discovered extralaryngeal terminal division show that this is a common anatomic variation. Therefore, surgeons frequently encounter bifurcated nerve during thyroidectomy operations. Previous manuscripts have also reported the usualness of this variation [1, 2, 4, 5]. The incidence of bifurcated nerve has been reported between 18% and 43% [6–11]. Sometimes, we can unusually find three terminal branches of the ILN in its cervical course. Our incidence (2% of ILNs) of trifurcated nerve shows that ILN is rarely divided three terminal branches prior to its laryngeal entry. The mean incidence of terminal trifurcation of the ILN has also been reported approximately 2% (between 1% and 3.8%) in previous studies [6, 8, 10, 12]. The incidence of extralaryngeal terminal division of the ILN is high in which surgeons must strongly take into account the presence of this variation and be cautious exposing the ILN. 

Terminal division of the nerve may occur unilaterally or bilaterally at both sides. We generally observed unilateral bifurcation of the ILN in which 75% of our patients have got unilateral variation. Bilateral terminal division of the ILN is relatively uncommon. Bilaterality of this variation has been observed in 11% of our thyroidectomy cases and in 25% of patients with bifurcated nerves. Extralaryngeal bilateral division of the nerve has been reported in a large range of incidence between 3, to 33% [6, 7, 9–11, 13]. In our series, the left ILN (35%) is more commonly bifurcated than the right one (19%). The report of Makay et al. [12] confirms this finding of higher rate at the left side. Based on anatomical knowledge, this is an expected result. The left nerve recurrent under aortic arch is longer than the right one which is recurrent under right subclavian artery. Therefore, extralaryngeal division of the longer nerve is more commonly expected than the shorter one. On the other hand, the majority of previous studies have reported equal or slightly higher incidence at the right side. The rate has been found as 23, to 59% at the right and 10, to 49% at the left side [6, 7, 9–11, 13]. Branched ILNs may represent a risk factor for nerve palsy. Knowledge of this anatomical variation and its routine investigation are essential. Surgeon's awareness of bifurcated ILN at both sides is required for the safety of thyroid operations. 

In case of bifurcated ILN, anatomical location of division point along its cervical course is crucial in order to securely identify, to properly expose all branches of the nerve, and to protect nerve integrity without inadvertent injury to neural structures. In our study, we determined various locations of division point along the cervical course of the exposed ILN. Approximately in half (25/54) of the bifurcated nerves, the division occurs at or very close to nerve-artery crossing point. Bifurcation of the nerve just adjacent to neuro-vascular (the ILN and the ITA) crossing is the most common location of the division point of the ILN. The division of the nerve occurs distal to the ITA (after nerve-artery crossing) in approximately 1/3 of our cases in which this is found as the second common location along the cervical course of the nerve. Beside these two common findings, we also determined two other locations of the division point. The bifurcation of the nerve seldom occurs proximal to the ITA (before nerve-artery crossing). The division point is uncommonly observed very close to the laryngeal entry of the nerve. 

We try to classify anatomic location of division point of the ILN along its cervical course aiming to increase surgeon's awareness. In order to establish an understandable classification some anatomic structures and surgical variables are taken into account.

1. *Landmarks to delineate cervical segments along cervical course of the ILN*: two relatively constant landmarks (inferior thyroid artery and laryngeal entry point) help to divide cervical course of the nerve by four segments; pre-arterial, arterial, post-arterial, and pre-laryngeal.
2. *Surgical technique to identify and expose the ILN*: the ILN is identified at close proximity of ITA by standard lateral approach technique. The nerve dissection begins in arterial region, and is proximally and distally advanced towards laryngeal entry in order to expose the entire nerve.
3. *Rates of division of the nerve in various locations*: approximately half of nerve bifurcation occurs near ITA.

Therefore, anatomic landmarks, surgical technique of nerve identification and exposition, and location rates of division point contribute to the classification of extralaryngeal bifurcation. Extralaryngeal bifurcation of the ILN is a common variation which carries great importance for preservation of its motor function. We think that to be familiar with location of division point of the nerve in its cervical course is significantly helpful to avoid nerve injury. We propose an anatomical classification of extralaryngeal terminal division of the ILN. Terminal division of the nerve is classified by four types.

**Type 1**

The bifurcation occurs at or very close (−/+  5 mm) to nerve-artery crossing point. The identification and dissection of the nerve generally begins at this point by standard lateral approach. In experienced hands, the nerve is easily found adjacent to the artery. Therefore, this type of bifurcation is also easily identifiable with careful dissection. This location is the most common type of nerve division in our series. Both branches are individually followed until their separated laryngeal entry.

**Type 2**

After identification of the nerve trunk, the dissection is advanced in both distal and proximal direction in order to display entire cervical course of the ILN. In some patients, nerve bifurcation occurs distally to the artery, near Berry ligament. In our series, the rate of post-arterial (Type 2) division of the nerve is found approximately in one third of our patients with bifurcated nerve.

**Type 3**

During distal dissection of the ILN trunk, another type of bifurcation is discovered at the most distal part of the extralaryngeal ILN. Pre-laryngeal (Type 3) bifurcation occurs most distally just before laryngeal entry of the nerve. This distal part is generally covered by ligament of Berry and thyroidal tissue. This region where the nerve is more superficial is reported as the most dangerous site for nerve injury. If the bifurcation is not identified proximally (Types 1 and 2), surgeons are faced with a dangerous situation. Surgeons must be aware of this distal division of the ILN. If surgeons identify single trunk of the nerve at arterial region, the dissection is carefully advanced to distal part for possible pre-laryngeal bifurcation. This type of most distal bifurcation must take a strong place in surgeons mind in order to avoid a disaster after the operation. Fortunately, pre-laryngeal (Type 3) division of the nerve seldom occurs as 11% of our patients with bifurcated nerve.

**Type 4**

The dissection of the nerve is also advanced proximal to the artery along cervical course of the ILN. In some patients, bifurcation of the nerve is found at proximal segment of the nerve before artery crossing. Pre-arterial (Type 4) bifurcation which is found in 11% of our patients, is a relatively uncommon variation according to Types 1 and 2 division.

In case of bifurcation of the ILN, the location of motor fibers in nerve branches has extreme importance for preservation motor function. The motor fibers are located exclusively in the anterior branch, and the posterior branch is sensory in function [4, 7, 9, 11]. In some cases. posterior cricoarytenoid (PCA) muscles receive any kind of nerve fibers from posterior division of the ILN. These fibers contribute innervations of PCA muscle [14, 15]. On the other hand, Maranillo et al. [16] have reported that PCA muscles, the only abductor muscle of the larynx receive motor fiber from anterior division of the ILN.

Extralaryngeal terminal branches of the ILN can be a potential cause of nerve injury due to visual misidentification. This anatomical variation cannot be predicted preoperatively and might be associated with higher rate of nerve injury [17]. Extralaryngeal bifurcation of the ILN increases the risk of vocal cord palsy by two folds [18]. Recognition of bifurcation is crucial. Preservation of all branches is required for prevention of vocal cord palsy. We think that the most dangerous situation is misidentification and misinterpretation of relatively larger posterior branch as the main trunk of the nerve. In this situation, the anterior branch is under the greatest risk. Inadvertent division of motor fibers may lead to laryngeal muscles palsy despite the surgeon believing the nerve was preserved. In recent years intraoperative neural monitoring (IONM) assessing motor function of the nerve, helps surgeons to identify securely the ILN during thyroid surgery. IONM has gained widespread acceptance as an adjunct to visual nerve identification. Visual identification and a standard and uniform use of IONM facilitate to establish motor fibers of the nerve especially in high risk patients [19, 20]. Anatomical variations of the nerve including extralaryngeal division might be considered as high risk situations. Therefore, visual identification of the ILN and IONM may be extremely helpful for preserving motor function and preventing vocal cord palsy in case of bifurcated ILN.

## 5. Conclusion 

Extralaryngeal terminal division of the ILN is a common anatomical variation.

The bifurcation occurs in different segment of the ILN along its cervical course at various distances from cricothyroid membrane (laryngeal entry point). In the majority of such cases, the point of division is located near arterial junction (ILN and ITA crossing point). On the other hand, surgeons must take into account that there are also uncommon locations of the bifurcation. 

The anatomic classification of division point may increase surgeons' awareness of this variation. The classification may also lead to simplify identification and exposure of terminal branches.

 The ILN must be macroscopically identified and preserved during thyroidectomy in order to prevent vocal cord palsy. Identification, exposure, and preservation of both extralaryngeal terminal branches of the ILN possess paramount importance in order to prevent nerve injury. Misidentification and misinterpretation of the posterior branch as the main trunk of the ILN put the anterior branch and motor fibers under the greatest risk. Inadvertent division of motor fibers may lead to laryngeal muscles palsy despite the surgeon believing the nerve was preserved.

## Figures and Tables

**Table 1:** Classification of anatomical location of terminal division point of the ILN*.

Type	Region	Definition
1	Arterial	Division occurs at or closely adjacent (±5 mm) to neurovascular crossing of the ILN and the ITA*.

2	Postarterial	Division occurs in the first (proximal) half of the distance between neurovascular crossing and laryngeal entry point of the ILN.

3	Prelaryngeal	Division occurs in the second (distal) half of the distance between neurovascular crossing and laryngeal entry point of the ILN.

4	Prearterial	Division occurs before (proximal to) neurovascular crossing of the ILN and the ITA.

*ILN: inferior laryngeal nerve. ITA: inferior thyroid artery.

**Table 2:** The rate and lateral site of terminal division (bifurcation) of the inferior laryngeal nerve.

	Cases	Division	Bilateral division	Division at right side	Division at left side
Total thyroidectomy	100	43 (43)*	11	8	24

Inferior laryngeal nerve Trifurcation	200	54 (27) 4 (2)		19 (19) 2 (2)	35 (35) 2 (2)

Sex (Female/male)	77/23	33/10		15/4	27/8

*Numbers in parentheses are percentages.

**Table 3:** The incidence of terminal division of the ILN according to anatomical classification.

Type	Regions	Definition	Right	Left	Total
1	Arterial	Division occurs at or closely adjacent to neurovascular crossing.	9 (47.4)*	16 (45.7)	25 (46.3)
2	Postarterial	Division occurs in the first (proximal) half of the distance between neurovascular crossing and laryngeal entry.	7 (36.8)	10 (28.6)	17 (31.5)
3	Prelaryngeal	Division occurs in the second (distal) half of the distance between neurovascular crossing and laryngeal entry.	2 (10.5)	4 (11.4)	6 (11.1)
4	Prearterial	Division occurs before (proximal to) neurovascular crossing.	1 (5.3)	5 (14.3)	6 (11.1)

			19 (100)	35 (100)	54 (100)

*Numbers in parentheses are percentages.
